# A Novel Nitrobenzoate Microtubule Inhibitor that Overcomes Multidrug Resistance Exhibits Antitumor Activity

**DOI:** 10.1038/srep31472

**Published:** 2016-08-11

**Authors:** Yan-Bo Zheng, Jian-Hua Gong, Xiu-Jun Liu, Shu-Ying Wu, Yi Li, Xian-Dong Xu, Bo-Yang Shang, Jin-Ming Zhou, Zhi-Ling Zhu, Shu-Yi Si, Yong-Su Zhen

**Affiliations:** 1 Institute of Medicinal Biotechnology, Chinese Academy of Medical Sciences and Peking Union Medical College, Beijing 100050, P.R.China

## Abstract

Multidrug resistance is a major limitation for microtubule-binding agents in cancer treatment. Here we report a novel microtubule inhibitor (2-morpholin-4-yl-5-nitro-benzoic acid 4-methylsulfanyl-benzyl ester, IMB5046), its cytotoxicity against multidrug-resistant cell lines and its antitumor efficacy in animal models. IMB5046 disrupted microtubule structures in cells and inhibited purified tubulin polymerization *in vitro*. It bound to the colchicine pocket of tubulin. IMB5046 displayed potent cytotoxicity against multiple tumor cell lines with an IC_50_ range of 0.037–0.426 μM. Notably, several multidrug-resistant cell lines which were resistant to colchicine, vincristine and paclitaxel remained sensitive to IMB5046. IMB5046 was not a P-glycoprotein substrate. IMB5046 blocked cell cycle at G2/M phase and induced cell apoptosis. Microarray assay indicated that the differentially expressed genes after IMB5046 treatment were highly related to immune system, cell death and cancer. In a mouse xenograft model IMB5046 inhibited the growth of human lung tumor xenograft by 83% at a well-tolerated dose. It is concluded that IMB5046 is a tubulin polymerization inhibitor with novel chemical structure and can overcome multidrug resistance. It is a promising lead compound for cancer chemotherapy, especially for treatment of multidrug-resistant tumors.

Microtubules are highly dynamic cytoskeletal fibers composed of α- and β-tubulin heterodimers, and are involved in a variety of fundamental cellular processes, such as the maintenance of cell shape, intracellular trafficking, cell movement, and most recognized mitosis^1^,^2^. The fast dynamics of spindle microtubules make the rapidly dividing cells exquisitely sensitive to microtubule-binding agents^3^,^4^. These agents are broadly classified into two main groups: the microtubule-destabilizing agents (such as colchicine and vincristine), and the microtubule-stabilizing agents (such as paclitaxel and laulimalide). Given the success of taxanes and vinca alkaloids in the clinic, microtubule-binding agents represent one of the most active classes of drugs in the treatment of cancer^3^,^4^. However, resistance to these drugs is common^5^,^6^. The expression of P-glycoprotein (P-gp) efflux pump and different β-tubulin isotypes play important roles in the development of drug resistance^3^,^5^,^7^. As reported, both taxanes and vinca alkaloids are substrates of P-gp^8^. P-gp executes efflux of these drugs, and consequently reduces their intracellular concentrations and their cytotoxic activity^3^. Other limitations of microtubule-binding agents include neurological toxicity, poor solubility, cumbersome synthesis/purification, etc^4^,^6^,^9^. Searching for new microtubule inhibitors with novel chemical structure will help to overcome these limitations.

During the screening of anticancer drugs using the EMT-mimetic (epithelial-mesenchymal transition) assay, we found a highly active sample designated IMB5046. This was a nitrobenzoate compound, verified as 2-morpholin-4-yl-5-nitro-benzoic acid 4-methylsulfanyl-benzyl ester (Fig. 1a). Herein, we report its effect on microtubule assembly and its cytotoxicity against multidrug-resistant cell lines. Moreover, its antitumor efficacy against human tumor xenografts is also presented. To our knowledge, this is the first report that a nitrobenzoate compound can inhibit tubulin polymerization and overcome multidrug resistance.

## Results

### IMB5046 induces morphological changes and disrupts microtubule networks in cells

**Figure 1:**
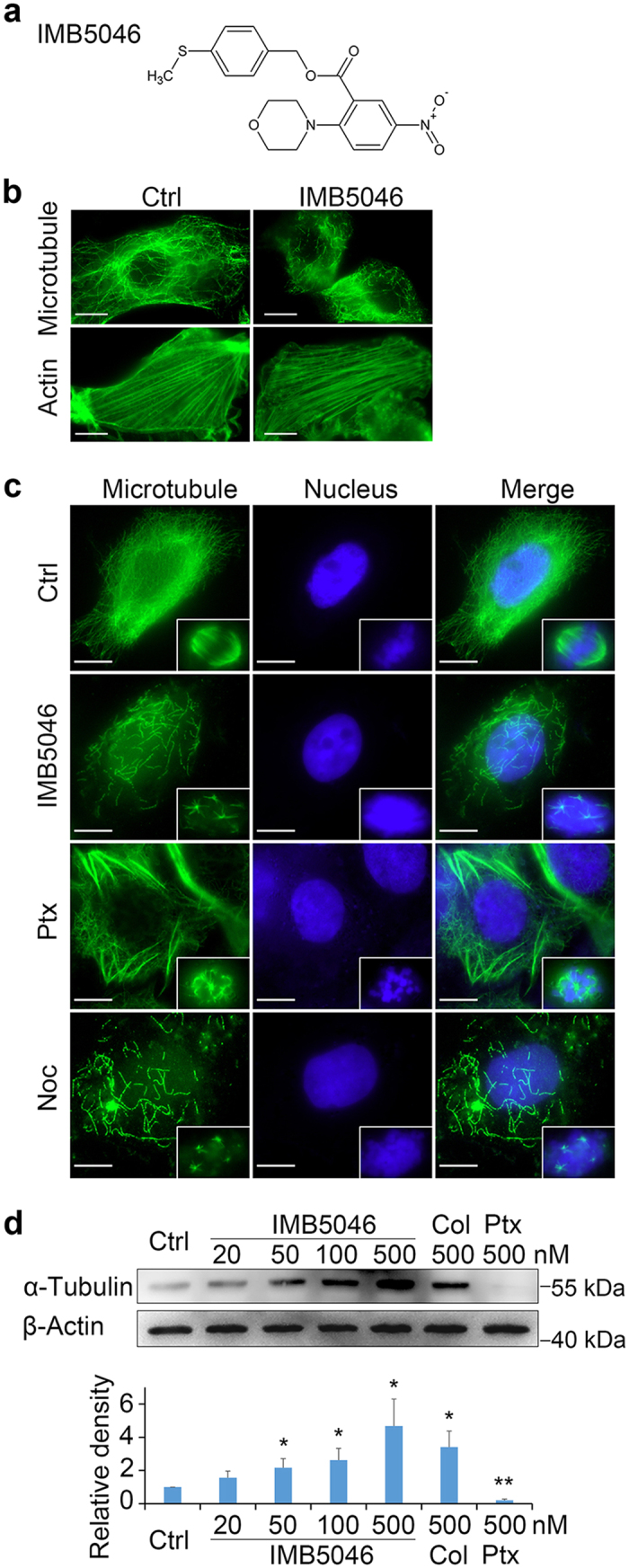
Chemical structure of IMB5046 and its effect on microtubules. (**a**) Chemical structure of IMB5046. (**b**) Effect of IMB5046 on the cytoskeleton of NIH/3T3 cells. NIH/3T3 cells were incubated with or without 100 nM IMB5046 for 6 h. Microtubule was stained with anti-tubulin antibody and F-actin was stained with phalloidin-FITC. IMB5046 at 100 nM partially disrupted the microtubule structures characterized by short microtubule fragments, but had no effect on F-actin networks. Scale bar, 10 μm. (**c**) IMB5046 disrupted the microtubule structures in A431 cells. A431 cells were treated with 100 nM IMB5046, 500 nM paclitaxel or 100 ng/mL nocodazole for 6 h. Insets are mitotic spindles from the same preparation. Scale bar, 10 μm. (**d**) Microtubule assembly assay in A431 cells. IMB5046 increased the free tubulin content in a concentration-dependent manner. Representative result of three independent experiments is shown. The histogram shows the relative density of tubulin. Data are presented as mean ± SD (n = 3). *P < 0.05, **P < 0.01 versus Ctrl. Ctrl, control; Noc, nocodazole; Col, colchcine; Ptx, paclitaxel.

In the primary EMT-mimetic screening, we found that IMB5046 induced morphological changes of NIH/3T3 cells in a time and concentration-dependent manner similar to that induced by nocodazole, a microtubule-destabilizing agent, with rounding up of the initially spindle-shaped cells (data not presented). Because of the pivotal role of the cytoskeleton in the maintenance of cell shape, the effects of IMB5046 on the cytoskeleton were investigated. As shown in Fig. 1b, 100 nM IMB5046 disrupted the microtubule structures in NIH/3T3 cells but had no effect on F-actin networks. Human epidermoid carcinoma A431 cells were also treated with 100 nM IMB5046 for 6 h, and stained with anti-α-tubulin antibody for the detection of microtubules. As shown in Fig. 1c, untreated cells contained well-organized microtubule networks and mitotic spindles, whereas IMB5046 treated cells showed short microtubule fragments and reduced microtubule density, similar to that induced by nocodazole. This compares to paclitaxel treatment which promoted tubulin polymerization characterized by thick microtubule bundles. Otherwise, IMB5046, nocodazole and paclitaxel induced aberrant mitotic spindles that were mostly multiple asters.

To determine the effect of IMB5046 treatment on the free tubulin content in cells, we performed a microtubule assembly assay. A431 cells were treated with IMB5046, colchicine or paclitaxel for 6 h. Then, soluble tubulin in the cytoplasm was separated and detected by Western blot. Figure 1d shows that IMB5046 increased the free tubulin content in a concentration-dependent manner. As controls, colchicine increased the free tubulin content, while paclitaxel clearly decreased it. These results demonstrate that IMB5046 depolymerizes cellular microtubules and induces an increase of free tubulin content in cells.

### IMB5046 inhibits tubulin polymerization *in vitro*

**Figure 2:**
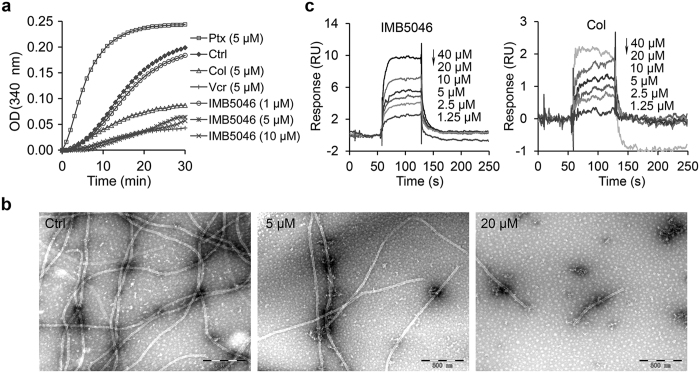
Inhibitory effects of IMB5046 on tubulin polymerization and its binding kinetics *in vitro*. (**a**) Tubulin polymerization assay *in vitro*. IMB5046 inhibited tubulin polymerization, evaluated by turbidity changes at a wavelength of 340 nm. The experiment was repeated twice. Data from representative experiment are shown. (**b**) Electron micrographs of microtubules assembled in the presence or absence of IMB5046. Negatively stained samples. Scale bar, 500 nm. (**c**) Binding kinetics of IMB5046 (1.25, 2.5, 5, 10, 20, 40 μM) and colchicine (1.25, 2.5, 5, 10, 20, 40 μM) to tubulin (2,000 RU) determined by SPR technology. The experiment was repeated twice. Data from representative experiment are shown. RU, Resonance Units.

As IMB5046 can disrupt the microtubule networks in cells, we tested whether IMB5046 could inhibit tubulin polymerization in a cell-free system. Purified tubulin was incubated with the drugs and the absorbance at 340 nm was recorded as an indicator of tubulin polymerization. The results showed that IMB5046 inhibited tubulin polymerization in a concentration-dependent manner with an IC_50_ of 2.97 μM (Fig. 2a). As controls, colchicine and vincristine inhibited tubulin polymerization, whereas paclitaxel enhanced polymerization (Fig. 2a).

Then the tubulin samples polymerized in the presence or absence of IMB5046 were analyzed by electron microscopy. As shown in Fig. 2b, the control sample showed typical microtubule polymers. While in the presence of 5 μM IMB5046, much fewer tubules were observed. In the presence of 20 μM IMB5046, only rare, short tubules were found. This result further demonstrated that IMB5046 strongly inhibited the polymerization of tubulin.

Surface plasmon resonance (SPR) technology was used to analyze the kinetic process of IMB5046/tubulin interaction. A SA sensor chip with streptavidin on the surface was used to immobilize biotin-tubulin. Then, IMB5046 or colchicine at different concentrations was injected over the sensor chip surface for binding detection. To both agents, the resonance units increased in a concentration-dependent manner (Fig. 2c). As determined by steady state fitting model, the equilibrium dissociation constant K_D_ of IMB5046 was 31.9 μM. For colchicine, the K_D_ value was 21.1 μM (Fig. 2c). These results demonstrate that IMB5046 can interact with tubulin directly and inhibit tubulin polymerization *in vitro*.

### IMB5046 binds to tubulin at the colchicine site

**Figure 3:**
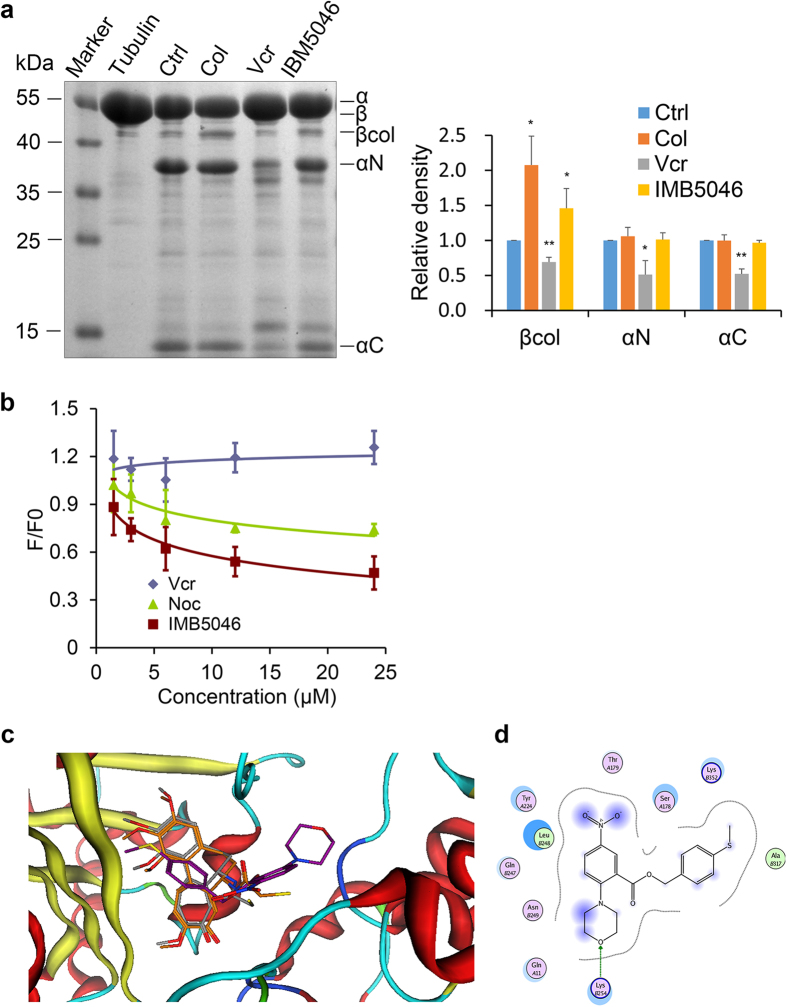
IMB5046 binds to tubulin at the colchicine pocket. (**a**) Limited proteolysis assay. Tubulin treated with IMB5046 showed similar patterns of proteolysis with that of colchicine. Representative result of three independent experiments is shown. The histogram shows the relative density of each fragment. Data are expressed as mean ± SD (n = 3). *P < 0.05, **P < 0.01 versus Ctrl. (**b**) Colchicine competition assay. IMB5046 and nocodazole decreased the intrinsic colchicine fluorescence, while vincristine did not. Data are expressed as mean ± SD (n = 3). (**c**) The superimposition of the predicted binding mode of colchicine (in stick, carbon in orange) with the crystal structure (in stick, carbon in grey), as well as the predicted binding mode of IMB5046 (in stick, carbon in purple). (**d**) The detailed interactions between IMB5046 and tubulin.

For microtubule-destabilizing agents, there are two established binding sites on tubulin, the vinca site and the colchicine site. A limited proteolysis assay is widely used to investigate the binding site of drugs on tubulin^10^,^11^. Binding of drugs to tubulin dimer will cause protein conformational changes, resulting in different patterns of proteolysis by trypsin. As shown in Fig. 3a, pre-incubation of tubulin with 100 μM IMB5046 followed by limited trypsin digestion produced an enhanced βcol fragment which was similar to that caused by colchicine. The vinca site agent vincristine substantially decreased the intensity of the βcol, αN and αC fragments. These results indicate that IMB5046 binds to tubulin at the colchicine site.

To further confirm the result, a colchicine competition assay was performed. As reported, competition between the test compound and colchicine for the binding site will decrease the intrinsic fluorescence of colchicine-tubulin complex by reducing the amount of colchicine bound^12^. As shown in Fig. 3b, IMB5046 decreased the intrinsic colchicine fluorescence in a concentration-dependent manner. As a positive control, nocodazole which is known to bind at the colchicine site decreased the fluorescence, while vincristine had no effect on the fluorescence. These results further support that IMB5046 binds to tubulin at the colchicine site.

A molecular modeling study was conducted to explore the interaction between tubulin and IMB5046. In a docking study, colchicine, the original ligand in the crystal structure, was selected as the reference compound, and the predicted binding mode generated through molecular docking was quite closed to that in the crystal structure, with an RMSD value of 0.50 Å (Fig. 3c), which indicates the docking results are reasonable. Therefore, IMB5046 was docked into the binding site of the catalytic domain in the same protocol, and the docking score of IMB5046 (−21.7 kcal/mol) was comparable to that of colchicine (−21.9 kcal/mol). In particular, IMB5046 forms a hydrogen bond interaction with Lys254, as well as hydrophobic interactions with Leu248, Ala317 of the β subunit at the site (Fig. 3d), exhibiting a different binding mode from colchicine, which mainly forms hydrophobic interactions with α and β subunits. Thus, IMB5046, with a novel scaffold, targets the colchicine-binding site of tubulin in a divergent mode, and may provide a type of promising lead compound.

### Cytotoxicity of IMB5046 against different parent and resistant tumor cell lines

The cytotoxicity of IMB5046 against a variety of tumor cell lines was tested using an MTT assay. Different cell lines showed varied sensitivities to IMB5046 (Table 1a). As shown, A431, HT-1080 and HT29 cells were highly sensitive to IMB5046 with an IC_50_ below 0.1 μM. By contrast, NIH/3T3 was relatively resistant to IMB5046 with IC_50_ of 10.22 μM.

The overexpression of P-gp will cause multidrug resistance. The P-gp overexpressing KB_V200_ cells were employed to evaluate the cytotoxicity of different microtubule-binding agents. As shown in Table 1b, IMB5046 showed similar potent cytotoxicity toward KB_V200_ cells and the parent sensitive KB cells with a resistance index of 1.4-fold. Whereas, KB_V200_ cells were highly resistance to vincristine (11.2-fold), colchicine (5.6-fold) and paclitaxel (5.6-fold) compared with parental KB cells.

The cytotoxicity of IMB5046 to another multidrug-resistant cell line MCF7/ADR was also tested. IMB5046 showed similar cytotoxicity toward MCF7/ADR and MCF7 cells with a resistance index of 1.1-fold. Whereas, MCF7/ADR cells displayed highly resistance to vincristine (139.9-fold), colchicine (60.9-fold) and paclitaxel (102.2-fold) relative to parental MCF7 cells (Table 1b).

To investigate whether IMB5046 is a substrate of P-gp, drug-stimulated activity of P-gp ATPase was examined. As presented in Table 1c, verapamil and vincristine induced an obvious increase in activity of P-gp ATPase and the wells showed relatively lower luminescent signals, reflecting lower ATP concentrations; however, IMB5046 exerted no stimulation of the P-gp ATPase activity and the wells showed stronger signals. These results indicate that IMB5046 is not a substrate of P-gp.

### IMB5046 caused G2/M arrest and induced apoptosis

**Figure 4:**
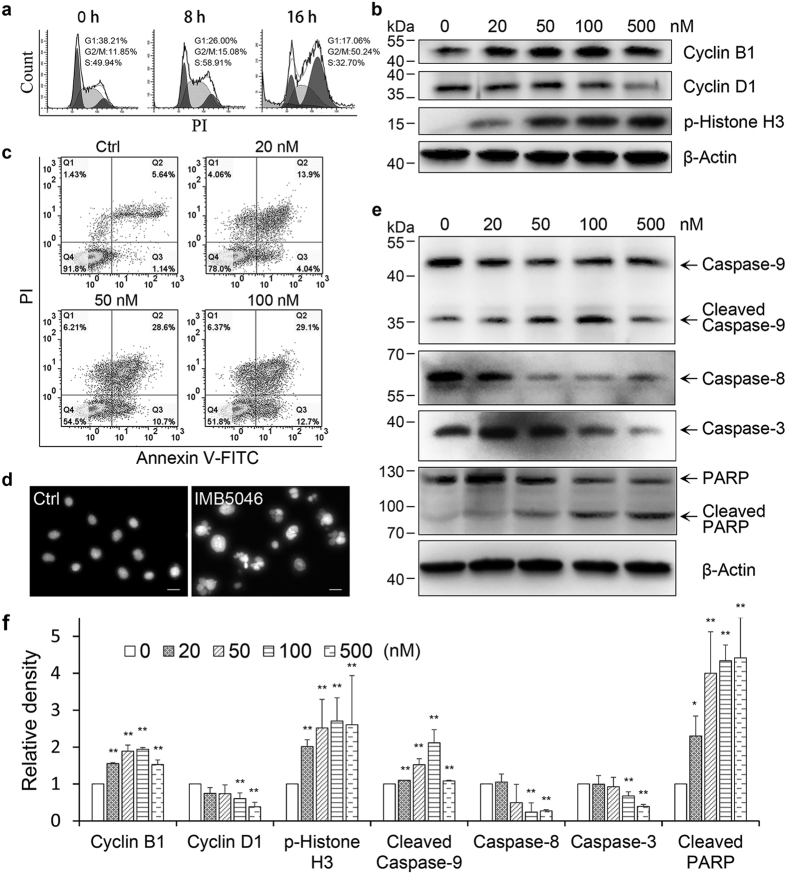
IMB5046 arrests cells at G2/M phase and induces apoptosis. (**a**) Cell cycle analysis by flow cytometry. IMB5046 arrests A431 cells in G2/M phase. (**b**) Western blot assay of cell cycle regulatory proteins after IMB5046 treatment. A431 cells were treated with IMB5046 at indicated concentrations for 24 h, then cyclin B1, cyclin D1 and p-Histone H3 were detected. Representative images of three independent experiments are shown. **(c)** Apoptosis assay by flow cytometry. IMB5046 induced apoptosis of A431 cells in a concentration-dependent manner. The lower-right quadrant shows the early apoptotic cells, the upper-right quadrant shows necrotic or late apoptotic cells, the upper-left quadrant shows necrotic cells or nuclear debris, and the lower-left quadrant shows healthy, viable cells. **(d)** Apoptotic bodies induced by IMB5046 treatment in A431 cells. Scale bar, 20 μm. **(e)** Western blot analysis of apoptosis-related proteins. A431 cells were treated with IMB5046 at indicated concentrations for 24 h, then caspase-3, 8, 9 and PARP were detected. Representative images of three independent experiments are shown. **(f)** The histogram shows the relative density of proteins shown in (**b**,**e**). Data are presented as mean ± SD (n = 3). *P < 0.05, **P < 0.01 versus control (0 nM).

The effect of IMB5046 on cell cycle progression was investigated by flow cytometry. As shown in Fig. 4a, IMB5046 induced G2/M arrest in a time-dependent manner in A431 cells. Accordingly, the G0/G1 cell population was decreased. In addition, a characteristic hypodiploid DNA content peak (sub-G1) appeared at 16 h treatment, indicating apoptotic cells. Furthermore, the effects of IMB5046 on the expression of cell cycle regulatory proteins were investigated using Western blot. As shown in Fig. 4b and f, treatment of A431 cells with IMB5046 resulted in up-regulation of cyclin B1, down-regulation of cyclin D1, and up-regulation of phosphorylated Histone H3 which is only seen in mitotic cells. These results provide further evidence of G2/M phase blockage by IMB5046.

The effect of IMB5046 on cell apoptosis was investigated with Annexin V-FITC/PI staining. Annexin V-positive and PI-negative cells represent early apoptotic cells, and double positive cells are defined as necrotic or late apoptotic cells. As shown in Fig. 4c, IMB5046 induced apoptosis of A431 cells in a concentration-dependent manner. Treatment of A431 cells with 50 nM IMB5046 for 24 h also induced a great number of apoptotic bodies (Fig. 4d). The involvement of cysteine-aspartic proteases (caspases) in IMB5046-induced apoptosis was investigated. As shown in Fig. 4e,f, treatment of A431 cells with IMB5046 induced the cleavage of executioner caspase-3 and its downstream substrate poly (ADP-ribose) polymerase (PARP). Caspase-8 and -9, the two major upstream activators of caspase-3, were activated (Fig. 4e,f).

### The gene expression profile analysis after IMB5046 treatment

**Figure 5:**
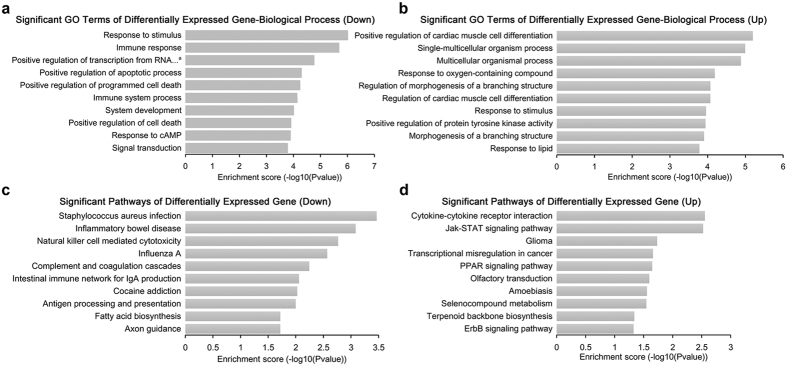
GO biological process and KEGG pathway analysis of the differentially expressed genes after IMB5046 treatment. (**a**) The top ten down-regulated terms in GO biological process classification. ^a^Positive regulation of transcription from RNA polymerase II promoter in response to stress. (**b**) The top ten up-regulated terms in GO biological process classification. (**c**) The top ten significant down-regulated pathways in KEGG pathway analysis. (**d**) The top ten significant up-regulated pathways in KEGG pathway analysis.

The whole human genome microarray was used to further analyze the differentially expressed genes in A431 cells after IMB5046 treatment. A total of 441 genes were down-regulated and 383 genes were up-regulated by at least 2-fold between IMB5046-treated and control cells. As shown in Fig. 5a, in the top ten significant down-regulated terms of GO (Gene Ontology) biological process, 3 were related to cell death or apoptosis, 2 to immune system. In the top ten up-regulated terms, 2 were related to morphogenesis, 2 to muscle cell differentiation (Fig. 5b). In the top ten down-regulated KEGG (Kyoto Encyclopedia of Genes and Genomes) pathways, 5 were related to immune system or inflammation, 2 to virus (Fig. 5c). Other down-regulated pathways included focal adhesion, regulation of actin cytoskeleton, cell adhesion molecules (CAMs) etc. In the top ten up-regulated pathways, 2 were related to cancer, others included the cytokine-cytokine receptor interaction, Jak-STAT signaling pathway, ErbB signaling pathway etc (Fig. 5d). These data indicated that the genes affected by IMB5046 were highly related to immune system, cell death and cancer.

### *In vivo* antitumor activity and toxicopathological examination

**Figure 6:**
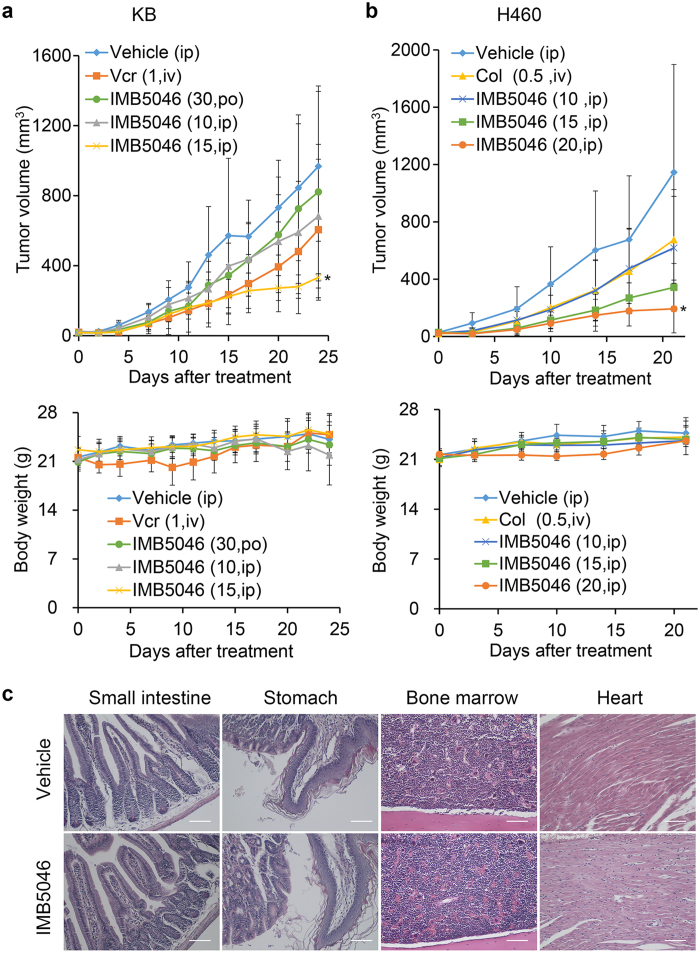
*In vivo* antitumor activity and toxicopathological examination. (**a**) Antitumor efficacy of IMB5046 in KB xenograft model and body weight of tumor-bearing mice during the experiment. IMB5046 (30, 10 and 15 mg/kg) and vehicle were given on days 0–4 & 7–11. Vincristine (1 mg/kg) was administrated on days 0 & 7. Data are presented as mean ± SD (n = 7). *P < 0.05 versus the vehicle group. (**b**) Antitumor efficacy of IMB5046 in H460 xenograft model and body weight of tumor-bearing mice. IMB5046 (10, 15 and 20 mg/kg) and vehicle were given on days 0–4 & 7–11. Colchicine (0.5 mg/kg) was administrated on days 0 & 7. Data are presented as mean ± SD (n = 5). *P < 0.05 versus the vehicle group. (**c**) Histopathological examination (hematoxylin and eosin stain) of mice treated with IMB5046 (20 mg/kg). No toxicological damage was found in the small intestine, stomach, bone marrow and heart. Scale bar, 50 μm.

*In vivo* antitumor efficacy of IMB5046 was evaluated in human epidermoid carcinoma KB model. At the end of the experiment, 13 days after the last administration, IMB5046 at i.p. (intraperitoneally) dose of 15 mg/kg inhibited the tumor growth by 65.6% which was obviously different from the vehicle group, whereas, IMB5046 at p.o. (orally) dose of 30 mg/kg just showed a marginal inhibitory rate (Fig. 6a). Vincristine at i.v. (intravenously) dose of 1 mg/kg inhibited the tumor growth by 37.4%. All treated groups showed no obvious body weight loss (Fig. 6a), or change in behavior during the experiment.

Therapeutic efficacy of IMB5046 was further evaluated in human lung cancer H460 xenograft model. IMB5046 at 10, 15 and 20 mg/kg (i.p.) inhibited tumor growth by 46.1%, 70.1% and 83.2%, respectively (Fig. 6b). Colchicine at 0.5 mg/kg (i.v.) inhibited the tumor growth by 41.1%. All treated groups showed no obvious body weight loss (Fig. 6b), or change in behavior during the experiment.

Colchicine was commonly toxic to the gastrointestinal tract, bone marrow, and heart etc^13^,^14^. In H460 xenograft model described above, histopathological examination of various organs of mice treated with IMB5046 (20 mg/kg) was performed. No histopathological lesions were found in the stomach, small intestine, bone marrow, and heart as well as other organs, such as the liver, kidney, lung, pancreas and spleen (Fig. 6c, Supplementary Fig. S1). These experiments indicated that the effective dose of IMB5046 was well tolerated.

## Discussion

Microtubules are key components of the cytoskeleton. We showed that IMB5046 disrupted the microtubule networks in cells, inhibited tubulin polymerization *in vitro* and interacted with tubulin dimer directly by binding at the colchicine pocket. All of these results demonstrate that IMB5046 is a tubulin polymerization inhibitor. IMB5046 has a novel chemical structure which is different from any known microtubule-binding agents. The new chemical structure confers it new properties. Though many of the microtubule-binding agents are the substrates of P-gp^7^, our experiment proved that IMB5046 was not a substrate of P-gp, showing similar potent cytotoxicity to both sensitive and resistant tumor cell lines. Otherwise, the small molecular weight (388 Dalton) and the relatively simple structure makes it easier to be synthesized. IMB5046 is a potential alternative agent for treatment of multidrug-resistant tumors.

We also noticed that after treatment with IMB5046, NIH/3T3 changed from a spindle-like shape to cobblestone–like shape, resembling the morphological changes that occur during the mesenchymal to epithelial transition (MET)^15^. It is reported that eribulin mesilate, a microtubule-depolymerizing drug, could induce MET and reverse EMT^16^,^17^. MET is usually accompanied by the reorganization of F-actin from cytoplasmic stress fibres to cortical bundles^18^. Whereas in our experiments, the IMB5046-treated cells still displayed strong stress fibres staining resembling those observed in the untreated cells. Whether IMB5046 can induce MET needs to be further studied.

Apoptosis can be triggered by death receptor-mediated extrinsic pathway or mitochondria-mediated intrinsic pathway. Most microtubule-binding agents induce apoptosis by the intrinsic mitochondrial-mediated pathway^7^,19–21. IMB5046 activated both caspase-9 and caspase-8. The activation of caspase-8 may have occurred downstream of the mitochondria, just serving as an executioner rather than an initiator of apoptosis^22^,^23^.

IMB5046 can obviously inhibit the growth of human tumor xenografts at a well-tolerated dose. In the mouse model, 15 mg/kg of IMB5046 inhibited the growth of KB xenografts by 65.6%. Though IMB5046 showed similar cytotoxity to both sensitive and resistant cell lines *in vitro, in vivo* antitumor activity of IMB5046 needs to be further evaluated using xenograft models established from the multidrug-resistant cell lines.

To further improve the antitumor efficacy, many new formulations of antimitotic agents have been developed and used in the clinic, such as nanoparticle albumin-bound paclitaxel (nab-paclitaxel)^24^,^25^,^26^ or antibody-conjugated microtubule-targeting agents such as brentuximab vedotin and trastuzumab emtansine^27^. In our experiments because of the poor aqueous solubility, IMB5046 was formulated with DMSO/cremophor/saline (1:2:17) and administrated intraperitoneally. To improve its solubility, reduce its side effects, and expand its therapeutic window, new IMB5046 formulations will be developed. Additionally, drug combination is also a hopeful strategy to overcome drug resistance and improve the efficacy of antimitotic agents^28^,^29^,^30^.

In the clinic, colchicine is used mainly for the treatment of gout and familial Mediterranean fever (FMF) due to its anti-inflammatory activities^31^. The result of genome microarray showed that the genes or pathways affected by IMB5046 were highly related to the immune system or inflammation, indicating that IMB5046 may also be useful in the treatment of diseases with inflammatory etiologies.

Conclusively, IMB5046 is a new tubulin polymerization inhibitor with the novel chemical structure of nitrobenzoate. Notably, it is not a substrate of P-gp pump and can overcome P-gp-mediated multidrug resistance. IMB5046 might provide hope for treatment of multidrug-resistant tumors.

## Methods

### Reagents and antibodies

IMB5046 (molecular weight, 388 Dalton; Fig. 1a) was synthesized with 2-morpholin-4-yl-5-nitrobenzoic acid and 4-(methylthio)benzyl alcohol in our laboratory with a purity over 99% (China Patent: No. 201510397191.4). Reagents: Vincristine (J&K Scientific Ltd.), Nocodazole (Sigma Aldrich), Colchicine (SERVA Feinbiochemica), Paclitaxel (Beijing Union Pharmaceutical Factory). Antibodies: mouse anti-α-Tubulin, anti-β-Actin, anti-PARP-1, and rabbit anti-p-Histone H3, anti-Cyclin D1, anti-Caspase-3 (Santa Cruze Biotechnology); mouse anti-Cyclin B1, anti-Capase-8, anti-Capase-9 (Cell Signaling Technology); FITC-conjugated anti-Mouse IgG, HRP-conjugated anti-Mouse IgG, HRP-conjugated anti-Rabbit IgG (Zhongshan Inc.).

### Cells culture and cytotoxicity assay

Cell lines and their origins are listed in Table 1a. A-431, HT-29, A549 and PANC-1 cells were obtained from the Cell Center of the Institute of Basic Medical Sciences, Chinese Academy of Medical Sciences and Peking Union Medical College (CAMS & PUMC). HT-1080 and NCI-H460 cells were obtained from the Institute of Materia Medica, CAMS & PUMC. Huh7 cells were purchased from ATCC. KYSE150 cells were purchased from BioPike (China). All of the above cell lines were cultured in RPMI-1640 medium (Gibco BRL Inc.), authenticated using short-tandem repeat (STR) profiling and used within 6 months. NIH/3T3 cells were obtained from Beijing Institute for Cancer Research and cultured in DMEM medium (Gibco BRL Inc.). Human epidermoid carcinoma KB cells and KB-derived multidrug-resistant KB_V200_ cells were cultured in RPMI-1640 medium without or with 200 nM vincristine^32^,^33^,^34^. Human breast carcinoma MCF7 cells and MCF7-derived multidrug-resistant MCF7/ADR cells were cultured in DMEM medium without or with 1 μg/mL adriamycin (ADR)^33^,^34^. Resistant cell lines were cultured in drug-free medium for 3 days before use. The RPMI-1640 or DMEM basal medium were supplemented with 10% fetal bovine serum (Gibco BRL Inc.), 100 μg/mL streptomycin and 100 U/mL penicillin. The cell cultures were maintained at 37 °C in a humidified atmosphere containing 5% CO_2_.

For determination of cytotoxicity, MTT assay was performed as previously reported^35^. Tumor cells were incubated with drugs for 48 h before MTT was added. Each sample contained three replicate wells.

### Immunofluorescence staining

Cells grown on coverslips were treated with IMB5046, paclitaxel or nocodazole, respectively, for 6 h. After fixation with 4% paraformaldehyde and permeabilization with 0.1% Triton X-100, the cells were incubated with anti-α-tubulin antibody for 60 min at 37 °C. After washing thrice, FITC-conjugated secondary antibody was added and incubated for 30 min. Then, the cells were treated with Hoechst33342 (10 μg/mL) for 10 min for DNA staining. For F-actin staining, the fixed cells were stained with 50 μg/mL phalloidin-FITC (Sigma Aldrich) for 40 min. The images were observed and collected under a fluorescence microscope (Olympus IX81).

### Microtubule Assembly Assay in cells

A431 cells were treated with drugs for 6 h, then lysed in lysis buffer (20 mM Tris-HCl, 1 mM MgCl_2_, 2 mM EGTA, 0.5% NP-40, 1 mM orthovanadate, 1 mM PMSF, and protease inhibitor cocktail Set III (Millipore), pH 6.8). After centrifugation at 12,000 rpm for 10 min at 4 °C, supernatants containing free tubulin were collected and the polymerized microtubules were discarded in the pellet. The supernatants were subjected to 12% SDS-PAGE and Western blot assay as previously reported^36^. Anti-α-tubulin antibody was used to visualize free tubulin. The experiment was repeated three times.

### Tubulin polymerization assay *in vitro*

The experiment was performed according to a HTS-Tubulin Polymerization Assay Kit (Cytoskeleton, Denver). Briefly, 4 mg/mL tubulin in G-PEM (80 mM PIPES, 2 mM MgCl_2_, 0.5 mM EGTA, pH 6.9) plus 10% glycerol buffer was mixed with test compounds in 96-well plates. The increase of absorbance at 340 nm was recorded every 30 s for 30 min at 37 °C in an Enspire 2300 multilabel reader (Perkin Elmer, MA, USA). The experiment was repeated twice, one sample for one concentration at each experiment.

### Electron microscopy

Tubulin was polymerized with or without IMB5046 at 37 °C for 30 min as described above. Then the samples were fixed with 0.5% glutaraldehyde for 5 min, applied to carbon-coated grids (300 mesh, Beijing Zhongjingkeyi Technology Co., Ltd) and negatively stained with 3% uranyl acetate. Samples were viewed using a transmission electron microscope (JEM 1200EX, JEOL, Japan).

### Surface plasmon resonance assay

Binding affinity with tubulin was analyzed using SPR technology in a Biacore T200 system (GE Healthcare Life Sciences). A Series S Sensor Chip SA (GE Healthcare Life Sciences) was preconditioned with three consecutive 1-min injections of 1 M NaCl in 50 mM NaOH. Then 50 μg/mL biotin-tubulin (Cytoskeleton, Denver) was immobilized to the sensor chip surface to attain 2,000 RU (1,000 RU correspond to an angle change of ~0.1°). One of the four flow cells on the chip was left free as a negative control. IMB5046 or colchicine at different concentrations was injected over the sensor chip surface for association analysis, followed by dissociation analysis. The experiment was repeated twice and all data were obtained at 25°C with running buffer HBS-EP (10 mM HEPES, 150 mM NaCl, 3.4 mM EDTA, and 0.005% (v/v) surfactant P20, pH 7.4). The equilibrium dissociation constant (K_D_) was calculated by steady state fitting mode with Biacore T200 Evaluation Software, version 2.

### Limited proteolysis assay

Limited proteolysis of tubulin and tubulin-drug complexes with trypsin was performed in MES buffer (0.1 M morpholinoethanesulfonic acid, 1 mM MgCl_2_, 1 mM EGTA, pH 6.9). Tubulin (1 mg/mL) was preincubated with 100 μM IMB5046, colchicine or vincristine for 30 min at 30 °C. Then, 25 μg/mL trypsin (TPCK-treated, Sigma Aldrich) was added (1:40, w/w to tubulin), and digested for 10 min on ice. After that, 0.01 mM leupeptin (Sigma Aldrich) was added to stop the reaction. Samples were then electrophoresed on 15% SDS-PAGE and stained with coomassie brilliant blue R250. The image was captured using image analysis system AIO Inc. The experiment was repeated three times. The values of band density were measured by densitometry with Image J software (NIH Image) and normalized to the control expressed as 1.

### Colchicine competition assay

Tubulin (3 μM) was mixed with colchicine (3 μM) and incubated with various concentrations of IMB5046, nocodazole or vincristine, respectively, in PEM buffer (80 mM PIPES, 2 mM MgCl_2_, 0.5 mM EGTA, pH 6.9) at 37 °C for 1 h. Fluorescence readings (ex/em 365/435 nm) were taken using an Enspire 2300 multilabel reader (Perkin Elmer, MA, USA) and the values were normalized to the control.

### Molecular modeling

The DMA-colchicine bound tubulin crystal structure (PDB-ID: 1SA0) was selected for docking studies^37^. The binding mode for IMB5046 towards the colchicine-binding site of tubulin was generated through molecular docking using Molecular Operating Environment (MOE) version 2009.10 (http://www.chemcomp.com). In general, the docking was performed through the “DOCK” module in MOE using the alpha triangle placement method. Refinement of the docked poses was carried out using the Forcefield refinement scheme and scored using both the affinity dG and London dG scoring system. The pose with the highest docking score was returned for further analysis.

### Cell cycle and apoptosis analysis

The effect of IMB5046 on cell cycle was analyzed by flow cytometry using propidium iodide (PI) DNA staining. A431 cells were treated with 100 nM IMB5046 for 8 h or 16 h. After fixation in 70% ethanol at 4 °C overnight, cells were incubated with 50 μg/mL PI and 100 μg/mL RNase A for 30 min at room temperature. Stained cells were analyzed in a flowcytometer (Beckman Coulter).

Apoptosis was determined using an Annexin V-FITC Apoptosis Detection Kit (Beijing Biosea Biotechnology). After treatment with IMB5046 for 48 h, cells were harvested and resuspended in 1× binding buffer. Annexin-V FITC and PI were added and incubated for 15 min at room temperature in the dark. The ratio of apoptotic cells was determined by flow cytometry (Beckman Coulter).

### Preparation of protein samples and Western blot analysis

Cells were lysed in the lysis buffer (50 mM Tris-HCl, 150 mM NaCl, 0.02% NaN_3_, 0.1% SDS, 100 μg/mL PMSF, 1 μg/mL Aprotinin, 1% NP-40, 0.5% sodium deoxycholate, pH8.0). Protein concentration was quantified using a BCA protein assay kit (Pierce). SDS-PAGE and Western blot analysis were performed as previously reported^36^. The values of band density were measured by densitometry with Image J software (NIH Image). Then, the values were corrected for β-Actin density and normalized to the control expressed as 1. Western blot analysis was repeated at least three times.

### P-gp ATPase activity

Drug-stimulated activity of P-gp ATPase was detected by Pgp-Glo^TM^ assay systems (Promega). Mixed P-gp membranes and 25 mM MgATP with 100 μM Na_3_VO_4_ (a selective inhibitor of P-gp), 200 μM verapamil (positive control), 2 μM vincristine or 2 μM IMB5046, respectively, and incubated for 40 min at 37 °C. Each sample was repeated in triplicate. Then ATP Detection Reagent was added to stop the reaction and initiate luminescence. The plate was incubated at room temperature for 20 min to allow the luminescent signal to develop. The luminescence of the samples was detected in an Enspire 2300 multilabel reader (Perkin Elmer, MA, USA) and the plate was photographed under image analysis system AIO Inc.

### Microarrays and gene expression analysis

A431 cells were treated with 100 nM IMB5046 or vehicle (0.1% DMSO) for 24 h. Then, total RNA was extracted from cells using TRIzol reagent (Invitrogen). An Agilent Whole Human Genome Oligo Microarray (4 × 44 K) was used to investigate the changes in transcriptional profiles. The experiment was performed in KangChen Bio-tech Inc. (Shanghai, China) according to the Agilent One-Color Microarray-Based Gene Expression Analysis protocol. Genes with ≥2.0-fold change between two groups were identified as differentially expressed genes. GO biological process analysis (topGO, Adrian Alexa and Jorg Rahnenfuhrer) and KEGG pathway analysis (http://www.genome.jp/kegg/) were performed. The microarray data were accessible through Gene Expression Omnibus (accession number GSE78944).

### *In vivo* therapy study

In KB and H460 xenograft models, tumors were established and inoculated into female NIH *(nu/nu)* athymic mice (18–22 g) as previously reported^35^. In the KB model, after 7 days of tumor transplantation, mice (7 per group) were treated with vehicle mixture, 10 or 15 mg/kg IMB5046 intraperitoneally, or 30 mg/kg IMB5046 orally for 5 days/week for 2 consecutive weeks. IMB5046 was dissolved in a vehicle mixture of DMSO/cremophor/saline (1:2:17). Vincristine was administered intravenously at 1 mg/kg for 1 day/week for 2 weeks (maximum tolerated dose)^38^. In the H460 xenograft model, after 7 days of tumor inoculation, mice (5 per group) were treated with IMB5046 at 10, 15 and 20 mg/kg or vehicle intraperitoneally for 5 days/week for 2 consecutive weeks. Colchicine was administered intravenously at 0.5 mg/kg for 1 day/week for 2 weeks. Tumor size was determined as previously reported^36^.

### Histopathological examination

For histopathological examination, specimens from various organs were taken from the treated and the control athymic mice when the experiment ended. Paraffin-embedded tissue sections of 5 μm thickness were prepared and placed on APES-coated slides. After dewaxing and rehydration, the sections were stained with hematoxylin and eosin and examined under a light microscope (Leica Inc.).

### Ethics Statement

All animal experiments were carried out under approval of the Committee on the Ethics of Animal Experiments of the Institute of Medicinal Biotechnology, Chinese Academy of Medical Sciences (IMBF20060302). The study protocols comply with the recommendations in the Regulation for the Management of Laboratory Animals of the Ministry of Science and Technology of China.

## Additional Information

**How to cite this article**: Zheng, Y.-B. *et al*. A Novel Nitrobenzoate Microtubule Inhibitor that Overcomes Multidrug Resistance Exhibits Antitumor Activity. *Sci. Rep.* **6**, 31472; doi: 10.1038/srep31472 (2016).

## Supplementary Material

Supplementary Information

## Figures and Tables

**Table 1:**
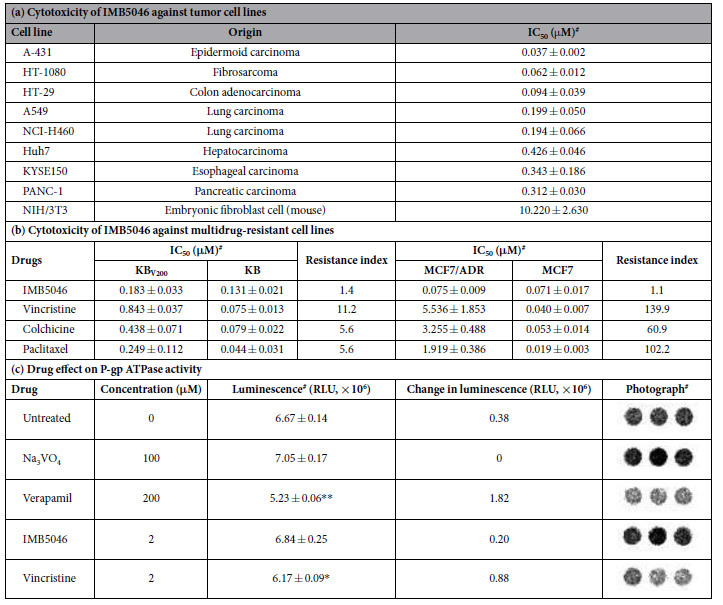
Cytotoxicity of IMB5046 against various cell lines and its effect on P-gp ATPase activity.

**P < 0.01, *P < 0.05 versus the untreated group.^#^Three repeats.
