# Supplementary material for: A Novel Nitrobenzoate Microtubule Inhibitor that Overcomes Multidrug Resistance Exhibits Antitumor Activity
Source: Sci Rep. 2016 Aug 11;6:31472. doi: 10.1038/srep31472 (PMC4980604; doi:10.1038/srep31472)
Supplement: Supplementary Information [file srep31472-s1.pdf]

A Novel Nitrobenzoate Microtubule Inhibitor that Overcomes Multidrug Resistance Exhibits Antitumor Activity

Yan-Bo Zheng, Jian-Hua Gong, Xiu-Jun Liu, Shu-Ying Wu, Yi Li, Xian-Dong Xu, Bo-Yang Shang, Jin-Ming Zhou, Zhi-Ling Zhu, Shu-Yi Si & Yong-Su Zhen*

Institute of Medicinal Biotechnology, Chinese Academy of Medical Sciences and Peking Union Medical College, Beijing 100050, P.R.China.

***Corresponding author:** Yong-Su Zhen

E-mail: zhenys@imb.pumc.edu.cn

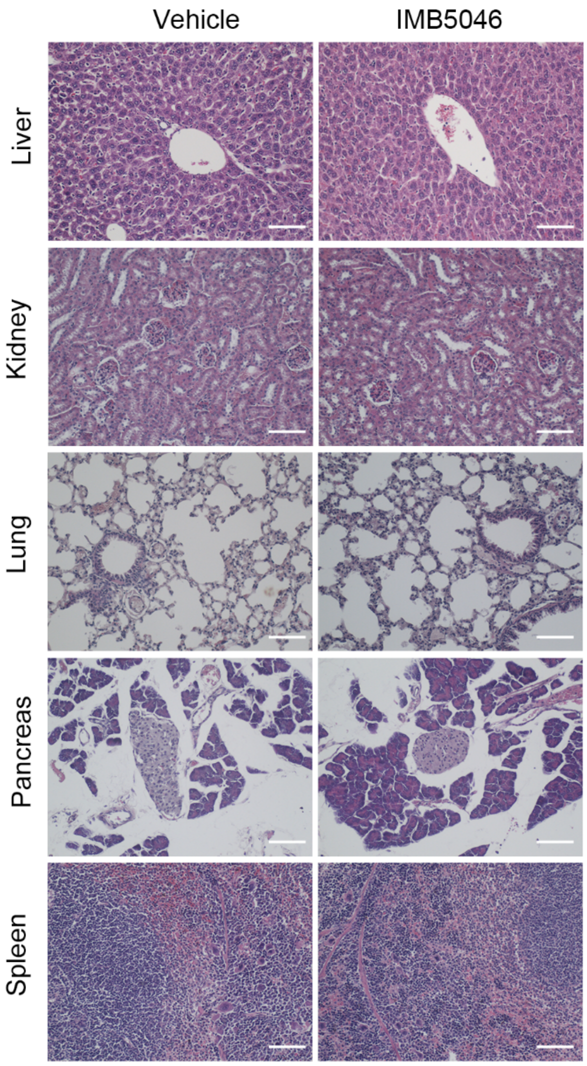

Supplementary Figure S1. Histopathological examination of mice treated with IMB5046. No histopathological changes were found in liver, kidney, lung, pancreas and spleen (hematoxylin and eosin stain). Scale bar, 50 μm.
